# Sharing Circulating Micro-RNAs between Osteoporosis and Sarcopenia: A Systematic Review

**DOI:** 10.3390/life13030602

**Published:** 2023-02-21

**Authors:** Francesca Salamanna, Deyanira Contartese, Alberto Ruffilli, Francesca Barile, Daniele Bellavia, Laura Marchese, Marco Manzetti, Giovanni Viroli, Cesare Faldini, Gianluca Giavaresi

**Affiliations:** 1 Surgical Sciences and Technologies, IRCCS Istituto Ortopedico Rizzoli, 40136 Bologna, Italy; 2 1st Orthopaedic and Traumatologic Clinic, IRCCS Istituto Ortopedico Rizzoli, 40136 Bologna, Italy; 3 Dipartimento di Scienze Biomediche e Neuromotorie DIBINEM, University of Bologna, 40125 Bologna, Italy

**Keywords:** osteoporosis, sarcopenia, osteosarcopenia, microRNA, systematic review

## Abstract

**Simple Summary:**

Osteoporosis and sarcopenia are common geriatric syndromes among the elderly population. Their coexistence was recently defined as osteosarcopenia, showing an incidence of ~37% in older adults, thus posing a serious global health burden. Thus, the search for osteosarcopenia biomarkers is mandatory for the early detection and prevention of deterioration of the condition. In this context, circulating microRNAs (miRs) show promise as advanced biomarkers. Here, we carried out a systematic review to explore and analyze the potential clinical biomarker utility of circulating miRs (serum, plasma, blood) shared between osteoporosis/osteopenia and sarcopenia.

**Abstract:**

Background: Osteosarcopenia, a combination of osteopenia/osteoporosis and sarcopenia, is a common condition among older adults. While numerous studies and meta-analyses have been conducted on osteoporosis biomarkers, biomarker utility in osteosarcopenia still lacks evidence. Here, we carried out a systematic review to explore and analyze the potential clinical of circulating microRNAs (miRs) shared between osteoporosis/osteopenia and sarcopenia. Methods: We performed a systematic review on PubMed, Scopus, and Embase for differentially expressed miRs (*p*-value < 0.05) in (i) osteoporosis and (ii) sarcopenia. Following screening for title and abstract and deduplication, 83 studies on osteoporosis and 11 on sarcopenia were identified for full-text screening. Full-text screening identified 54 studies on osteoporosis, 4 on sarcopenia, and 1 on both osteoporosis and sarcopenia. Results: A total of 69 miRs were identified for osteoporosis and 14 for sarcopenia. There were 9 shared miRs, with evidence of dysregulation (up- or down-regulation), in both osteoporosis and sarcopenia: miR-23a-3p, miR-29a, miR-93, miR-133a and b, miR-155, miR-206, miR-208, miR-222, and miR-328, with functions and targets implicated in the pathogenesis of osteosarcopenia. However, there was little agreement in the results across studies and insufficient data for miRs in sarcopenia, and only three miRs, miR-155, miR-206, and miR-328, showed the same direction of dysregulation (down-regulation) in both osteoporosis and sarcopenia. Additionally, for most identified miRs there has been no replication by more than one study, and this is particularly true for all miRs analyzed in sarcopenia. The study quality was typically rated intermediate/high risk of bias. The large heterogeneity of the studies made it impossible to perform a meta-analysis. Conclusions: The findings of this review are particularly novel, as miRs have not yet been explored in the context of osteosarcopenia. The dysregulation of miRs identified in this review may provide important clues to better understand the pathogenesis of osteosarcopenia, while also laying the foundations for further studies to lead to effective screening, monitoring, or treatment strategies.

## 1. Introduction

**Figure 1:**
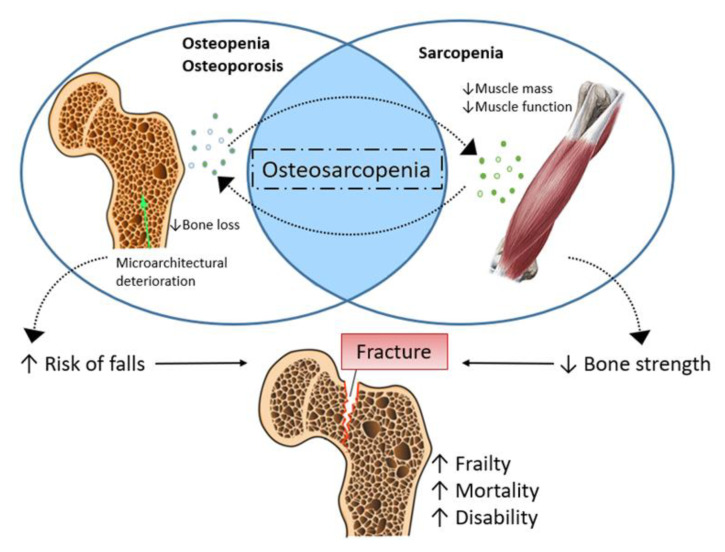
Schematic representation of osteosarcopenia.

Worldwide, the population of people over the age of 60 is expected to grow from 841 million in 2013 to more than 2 billion by 2050, with a percentage increase from 11 to 22% [1]. Unfortunately, this increase does not reflect an increase in ‘healthy life’ expectancy, and musculoskeletal aging is one of the most important health concerns [1]. Bone mass and muscle mass and strength start to reduce noticeably from the fifth decade of life [2]. Some evidence suggests that osteoporosis and sarcopenia have shared pathophysiological factors and common mechanical and molecular mechanisms [3,4,5,6]. Osteoporosis is described by deterioration in bone microarchitecture, resulting in decreased bone mineral density (BMD), increased bone fragility, and enhanced risk of fracture [7]. In contrast to osteoporosis, no one broadly accepted clinical definition of sarcopenia has yet been identified, although all definitions recognize that measuring muscle mass in isolation is inadequate, as a measure of muscle function is also required. An updated definition by the European Working Group on Sarcopenia in Older People in 2019 (EWGSOP2) gave a greater focus on low muscle strength as the primary parameter characterizing sarcopenia [8]. Recently, the coexistence of these two pathological conditions has been described and defined as ‘osteosarcopenia’, with the common denominator comprising age-related chronic inflammation (inflammaging), changes in body composition, and hormonal imbalance [9]. Its prevalence has been estimated at 10–15% in community-dwelling older adults, ~10% in those attending outpatient frailty clinics, and approximately 64% in osteoporosis outpatient clinics [10,11]. Osteoporosis and sarcopenia coexistence has been associated cross-sectionally with depression, malnutrition, peptic ulcer disease, inflammatory arthritis, and reduced mobility [10]. Several studies also revealed that individuals with both osteoporosis and sarcopenia are at higher risk of falls and fractures than those with osteoporosis or sarcopenia alone [10,11] (Figure 1). 

However, in contrast to osteoporosis and sarcopenia considered individually, to date, few data are available on osteosarcopenia. What is already known is that, considering the clinical outcomes linked with both osteoporosis and sarcopenia, the diagnosis of osteosarcopenia syndrome is mandatory for enabling clinical care [12]. The clinical diagnosis is hampered by three principal key difficulties in the evaluation of muscle and bone status [13,14]. First, despite imaging modalities such as dual-energy X-ray absorptiometry (DXA), magnetic resonance imaging (MRI), and computed tomography (CT) being able to provide an objective and appropriately estimation of body composition [13], these procedures are technically complex and commonly only available in well-equipped medical institutions. The use of bioelectrical impedance analysis (BIA), a potential tool for sarcopenia assessment, is of limited use in elderly individuals, since measured muscle mass may be underestimated due to inadequate hydration in aging populations [14]. Second, the repeatability of the estimation methods is inadequate. The main assessments for muscle function include usual gait speed and a short physical performance battery (SPPB) [14]. Third, osteoporosis and sarcopenia are chronic and multifactorial diseases, and not all individuals present the same rates of muscle and bone loss. Therefore, resultant indicators to track progression over time or response to specific interventions are critical. 

To overcome the absence of ‘gold standard’ techniques to correctly evaluate muscle and bone, several circulating biomarkers have been explored based on the molecular biological mechanisms of their involvement in the pathogenesis of sarcopenia and osteoporosis. Over the past few decades, a novel kind of RNA, microRNAs (miRs), have attracted great attention from researchers and clinicians as alternative and advanced biomarkers for numerous pathological conditions, leading to the conclusion that miRs are “fingerprints” for specific diseases [15,16,17,18,19,20]. MiRs are short, non-coding RNAs of typically 18–22 nucleotides that work as post-transcriptional regulators of protein-coding genes and the non-coding genome [21]. They are key molecular regulators in cells, which modify the expression of genes at a post-transcriptional level by impeding the translation of specific mRNAs or inducing specific mRNA degradation [21]. Significantly, mature miRs can exit cells and are detected in the bloodstream [20,21,22,23]. In 2008, two different research teams discovered and analyzed the presence of miRs in the bloodstream, and since then, various sequences have been found in human- and animal-derived plasma and serum [20,21,22,23]. However, to date, most miR studies have been conducted using cultured cells or animal model systems, and only a small number of studies have investigated changes in circulating miRs in pathological conditions such as osteoporosis and sarcopenia. Thus, considering the increasing prevalence of osteosarcopenia, the search for specific shared miRs between osteoporosis and sarcopenia should be considered mandatory for the early detection of the condition. The objective of this systematic review was to explore and analyze the potential clinical biomarker utility of circulating miRs (serum, plasma, blood) that are shared between osteoporosis/osteopenia and sarcopenia. To the best of our knowledge, there is no previous systematic review assessing shared miR between osteoporosis/osteopenia and sarcopenia.

## 2. Materials and Methods

### 2.1. Eligibility Criteria

The PICOS model (Population, Intervention, Comparison, Outcomes, Study design) was used to design this study: (1) studies that considered osteoporotic/osteopenic and sarcopenic patients (Population), submitted or not (2) to a specific surgical intervention (Interventions), (3) with or without a comparison group (healthy controls) (Comparisons), (4) that reported significant differences (*p* < 0.05) on specific circulating miRs (Outcomes), in (5) clinical studies (Study design). Studies from 2 January 2013 to 2 January 2023 were included in this review if they met the PICOS criteria. We excluded studies that evaluated (1) miRs in cells or animal model systems; (2) miRs in patients with other concomitant severe pathological conditions (e.g., cancer, metastases, diabetes, HIV, mastocytosis, thyroid pathologies, arthritis, acromegaly, ulcerative colitis, chronic heart failure, idiopathic and genetic osteoporosis, cerebral diseases) in addition to osteoporosis and sarcopenia; (3) miRs as modulators of drug resistance, in drug response and/or as drugs for medical intervention; (4) miRs for the constuction of mathematical modeling tools; (5) miRs variation in physical activity; (6) miRs transfection in cells; (7) miRs expression profiles in exosomes; (8) articles with incomplete outcomes or data. Additionally, we excluded reviews, letters, comments to editor, meta-analysis, editorials, protocols and recommendations, guidelines, and articles not written in English. 

### 2.2. Search Strategies

Our literature review involved a systematic search conducted in January 2023. We performed our review according to the Preferred Reporting Items for Systematic Reviews and Meta-Analyses (PRISMA) statement [24]. The search was carried out on three databases: PubMed, Scopus, and Embase. The following combination of terms was used: (osteoporosis) AND ((serum miR) OR (circulating miR)) and (sarcopenia) AND ((serum miR) OR (circulating miR)); for each of these terms, free words, and controlled vocabulary specific to each bibliographic database were combined using the operator “OR”. The combination of free-vocabulary and/or Medical Subject Headings (MeSH) terms for the identification of studies in PubMed, Scopus, and Embase are reported in Table 1.

### 2.3. Selection Process 

After submitting the articles to a public reference manager (Mendeley Desktop 1.19.8) to eliminate duplicates, possible relevant articles were screened using title and abstract by two reviewers (FS and DC). Studies that did not meet the inclusion criteria were excluded from review, and any disagreement was resolved through discussion until a consensus was reached or with the involvement of a third reviewer (GG). Subsequently, the remaining studies were included in the final stage of data extraction.

### 2.4. Data Collection Process and Synthesis Methods

The data extraction and synthesis process started with cataloging study details. To increase validity and avoid omitting potentially findings for the synthesis, two authors (FS and DC) extracted and constructed the tables (Table 2, Table 3, Table 4 and Table 5) while taking into consideration demographics data (country of publication, study design, patients number, ethnicity, sex/age, comorbidities, osteoporosis or sarcopenia diagnostic measures) and methodology of studies on osteoporosis and sarcopenia (miR, miR assay, tissue, endogenous genes, technical replicates, timing of sample collection, miR direction in osteoporosis or sarcopenia, main results).

### 2.5. Risk of Bias Assessment 

The methodological quality of selected studies was independently assessed by two reviewers (FS and DC), using the Quality Assessment of Diagnostic Accuracy Studies-2 (QUADAS-2) tool, which includes four risks of bias domains including “patient selection”, “index test”, “reference standard”, and “flow and timing” (flow of patients through the study and timing of the index tests and reference standard) [25]. Each domain is assessed in terms of high-, low-, or unclear risk of bias, and the first three domains are also assessed in terms of high-, low-, or unclear concerns about applicability [25]. In case of disagreement, the reviewers attempted to reach consensus by discussion; if this failed, a third reviewer (GG) was consulted to make the final decision.

**Table 2:** Demographics data on osteoporosis.

Ref.	Country of Publication	Study Design	Patients Number	Ethnicity	Sex/Age	Comorbidities	Osteoporosis Diagnostic Measures
Al-Rawaf 2021 [26]	Saudi Arabia	Prospective	100 Osteoporotic (N = 55) Healthy controls (N = 45)	NR	Female 50–80 years	None	DXA
Baloun 2022 [27]	Czechia	Prospective	22 After oophorectomy and hysterectomy (N = 11) Before oophorectomy and hysterectomy (N = 11)	NR	Female	NR	DXA
Bedene 2016 [28]	Slovenia	NR	74 Osteoporotic (N = 17) Healthy controls (N = 57)	NR	Female	NR	DXA FRAX
Chen 2016 [29]	China	NR	NR	Patients from Peking Union Medical College Hospital	Female	None	DXA
Chen 2017 [30]	China	NR	60 Osteoporotic (N = 30) Healthy controls (N = 30)	Chinese women	Female Osteoporotic: 59–80 years Non-osteoporotic: 62–74 years	None	NR
Chen 2019 [31]	China	NR	84 Osteoporotic (N = 42) Healthy controls (N = 42)	NR	Female	NR	NR
Chen 2019 b [32]	USA	NR	75 Osteoporotic/osteopenic (N = 46) Sarcopenic (N = 1) Sarco-osteopenic (N = 15) Non-osteoporotic/non-sarcopenic (N = 13)	NR	Female 60–85 years	None	DXA
Cheng 2019 [33]	China	NR	60 Osteoporotic (N = 30) Healthy controls (N = 30)	NR	Female	NR	NR
Ciuffi 2022 [34]	Italy	Prospective multicenter study	213 Osteoporotic (N = 105) Osteopenic (N = 62) Healthy controls (N = 46)		Female/male Osteoporotic 68.0 ± 4.9 years Osteoporotic with fragility fracture 68.6 ± 5.0 years Osteoporotic without fragility fracture 67.0 ± 4.5 years Osteopenic healthy controls 67.2 ± 5.0 years	NR	DXA
Ding 2019 [35]	China	NR	240 Osteoporotic (N = 120) Healthy controls (N = 120)	Chinese woman	Female	NR	NR
Feurer 2019 [36]	France	Prospective	682 Post-menopausal women (N = 583) Pre-menopausal women (N = 99)	NR	Female	Stage 4 renal failure (n = 2), hyperthyroidism (n = 5), rheumatoid arthritis (n = 3), diabetes (n = 18),	DXA HRpQCT
Fu 2019 [37]	China	Prospective	40 Osteoporotic (N = 20) Healthy controls (N = 20)	NR	Female	NR	NR
Fu 2021 [38]	China	Prospective	161 Osteoporotic (N = 82) Healthy controls (N = 79)	NR	Female/male Osteoporotic (60 female, 22 male) 50.48 ± 3.5 years Healthy controls (58 female, 21 male) 49.68 ± 4.17 years	NR	DXA
Gao 2020 [39]	China	NR	NR	NR	NR	NR	NR
Guo 2022 [40]	China	NR	40 Osteoporotic fractured patients (N = 20) Healthy controls (N = 20)	NR	Female Osteoporotic 59–80 years Healthy controls 62–75	NR	NR
Ismail 2020 [41]	Egypt	Prospective pilot	140 Osteoporotic (N = 70) Healthy controls (N = 70)	NR	Female Premenopausal (control: 34.03 ± 5.72 years and osteoporotic: 36.00 ± 7.15 years) Postmenopausal (control: 60.06 ± 6.57 and osteoporotic: 61.29 ± 7.69)	None	DXA
Li 2014 [42]	China	Prospective	120 Osteoporotic (N = 40) Osteopenic (N = 40) Healthy controls (N = 40)	Chinese woman	Female Osteoporotic 57.5 ± 11.3 years Osteopenic 56.7 ± 10.7 Healthy controls 56.5 ± 10.5	None	DXA
Li 2018 [43]	China	NR	20 Osteoporotic (N = 10) Healthy controls (N = 10)	Chinese woman	Female Age range 62–75 years	None	DXA
Li 2020 [44]	China	NR	72 Osteoporotic (N = 36) Healthy controls (N = 36)	NR	Female Osteoporotic 62 ± 3.98 years Healthy controls 59 ± 5.15 years	None	DXA
Lu 2021 [45]	China	NR	120 Osteoporotic (N = 63) Healthy controls (N = 57)	NR	Female Osteoporotic 49.97 ± 4.20 years Healthy controls 50.58 ± 4.14 years	None	DXA
Luo 2019 [46]	China	NR	NR	NR	NR	NR	NR
Lv 2019 [47]	China	Prospective	60 Osteoporotic (N = 30) Healthy controls (N = 30)	NR	NR	NR	NR
Ma 2021 [48]	China	Case-control	100 Osteoporotic (N = 86) Healthy controls (N = 14)	NR	Female Osteopenic/osteoporotic 65.00 ± 8.51 years Healthy controls 39.07 ± 2.87 years	None	DXA
Ma 2022 [49]	China	Case-control	158 Osteoporotic (N = 108; 58 with fragility fracture) Healthy controls (N = 50)	NR	Female Osteoporotic 64.82 ± 6.08 years Fragility fracture 63.72 ± 5.59 Healthy controls 64.26 ± 6.52 years	None	DXA
Mandourah 2018 [50]	United Kingdom	NR	Osteopenic without fracture (N = 63; F 53/M 10) Osteopenic with fracture (N = 15; F 13/M 2) Osteoporotic without fracture (N = 34; F 28/M6) Osteoporotic with fracture (N = 19; F 17/M 2) Healthy controls (N = 30; F 20/M 10)	NR	Female/male Osteopenic without fracture 65.6 ± 9.5 years Osteopenic with fracture 67 ± 9.5 years Osteoporotic without fracture 68.6 ± 10 years Osteoporotic with fracture 70 ± 10 years Healthy controls 67 ± 9.6 years	None	DXA
Mi 2020 [51]	China	NR	100 Osteoporotic (N = 50) Healthy controls (N = 50)	NR	Age range 53–74	NR	DXA
Nakashima 2020 [52]	Japan	Cross-sectional	352 Osteoporotic (N = 125) Healthy controls (N = 227)	Yakumo population	Female/male 64.1 ± 9.6 years	NR	DXA
Nobrega 2020 [53]	Brazil	Cross-sectional	40	Brazilian very old adults	Female and male 84.2 ± 4.5	Type-2 diabetes, hypertension, metabolic syndrome	DXA
Panach 2015 [54]	Spain	NR	25 Osteoporotic fractured (N = 14) Healthy controls (N = 11)	Caucasian women	Female Osteoporotic with fracture 79.6 ± 3.1 years Controls 63.4 ± 8.1 years	NR	DXA
Pertusa 2021 [55]	Spain	NR	77 Osteoporotic fractured (N = 25) Healthy controls (N = 52)	Caucasian women	Female Osteoporotic with fracture 79.6 ± 3.1 years Controls 76.8 ± 8.3 years	None	DXA
Qiao 2019 [56]	China	NR	100 Osteoporotic (N = 60) Healthy controls (N = 40)	NR	Female Osteoporotic 63.4 ± 2.4 years Healthy controls 59.3 ± 3.2 years	NR	DXA
Ramírez-Salazar 2019 [57]	Mexico	NR	87 Osteoporotic with fracture (N = 21) Osteoporotic without fracture (N = 16) Osteopenic (N = 28) Healthy controls (N = 22)	Mexican-Mestizo women	Female Osteoporotic 73.75 ± 4.46 years Healthy controls 71.1 ± 3.72 years	None	DXA
Salman 2021 [58]	Iraq	NR	95 Osteoporotic (N = 50) Healthy controls (N = 45)	NR	Female/male Osteoporotic 72.5 ± 9.45 years Healthy controls 71.4 ± 8.33 years	None	Physician diagnosis
Seeliger 2014 [59]	Germany	NR	60 Osteoporotic (N = 30) Healthy controls (N = 30)	NR	Female/male Osteoporotic 78.3 years Healthy controls 76.6 years	None	DXA, X-ray, qCT
Shuai 2020 [60]	China	Case-control	448 Osteopenia (N = 132) Osteoporotic (N = 134) Healthy controls (N = 182)	Northwest China	Female/male Osteopenia 49.0 years Osteoporosis 61.1 years Healthy controls 42.3 years	None	DXA
Sun 2020a [61]	China	NR	18 Osteoporotic with fracture (N = 6) Osteoporotic without fracture (N = 6) Healthy controls (N = 6)	NR	Female/male Osteoporotic without fracture 68.0 years Osteoporotic with fracture 69.7 years Healthy controls 47.8 years	None	DXA
Sun 2020b [62]	China	NR	81 Osteoporotic (N = 41) Healthy controls (N = 40)	NR	Female/male Osteoporotic with fracture 44 years	None	NR
Tang 2019 [63]	China	NR	30 Osteoporotic (N = 15) Healthy controls (N = 15)	NR	Female Age range 54–64	NR	NR
Wang 2018 [64]	China	NR	60 Osteoporotic (N = 45) Healthy controls (N = 15)	NR	NR	NR	NR
Weilner 2015 [65]	Austria	NR	23 Osteoporotic fractured (N = 12) Healthy controls (N = 11)	White Caucasian	Female age ≥ 65 years	Type-2 diabetes	DXA
Wu 2021 [66]	China	NR	20 Osteoporotic (N = 10; 6 females and 4 males) Healthy controls (N = 10; 5 females and 5 males)	NR	Female and male Osteoporotic Range 56–73 years Healthy controls Range 57–72 years	None	DXA
Xia 2018 [67]	China	NR	120 Osteoporotic (N = 60) Healthy controls (N = 60)	NR	Female	NR	qCT
Xu 2022 [68]	China	Retrospective	160 Osteoporotic patients with vertebral fractures (N = 78) Osteoporotic patients without vertebral fractures (N = 82)	NR	Osteoporotic patients with vertebral fractures 67.90 ± 7.04 years Osteoporotic patients without vertebral fractures 66.84 ± 6.58 years	None	DXA
Yang 2019 [69]	China	NR	30 Osteoporotic (N = 15) Healthy controls (N = 15)	NR	NR	NR	NR
Yavropoulou 2017 [70]	Greece	Multicenter cross-sectional, observational	100 Osteoporotic patients with vertebral fractures (N = 35) Osteoporotic patients without vertebral fractures (N = 35) Healthy controls (N = 30)	NR	Female Osteoporotic patients with vertebral fractures 71 ± 7 years Osteoporotic patients without vertebral fractures 68 ± 7 years Healthy controls 68 ± 5 years	None	DXA
Yin 2022 [71]	China	NR	95 Osteoporotic (N = 52) Healthy controls (N = 43)	NR	NR	None	NR
You 2016 [72]	China	NR	155 Osteoporotic (N = 81) Healthy controls (N = 74)	NR	Female Osteoporotic 65.8 ± 1.9 years Healthy controls 43.3 ± 1.4 years	NR	DXA
Yu 2020 [73]	China	NR	80 Osteoporotic with fracture (N = 40) Healthy controls (N = 40)	NR	Female/male Osteoporotic with fracture 60.8 ± 1.9 years Healthy controls 62 ± 2.5 years	None	DXA
Yuan 2021 [74]	China	NR	89 Osteoporotic (N = 47) Healthy controls (N = 42)	NR	NR	None	DXA
Zarecki 2020 [75]	United Kingdom	Case-control, observational, cross-sectional	107 Osteoporotic patients with vertebral fractures (N = 26) Osteoporotic patients without fractures (N = 39) Healthy controls (N = 42)	NR	Osteoporotic patients with vertebral fractures 69.6 years Osteoporotic patients without vertebral fractures 67.9 years Healthy controls 68.8 years	None	DXA
Zhang 2019 [76]	China	NR	Osteoporotic patients Healthy controls	NR	NR	None	NR
Zhang 2021 [77]	China	NR	116 Osteoporotic with fracture (N = 60) Healthy controls (N = 56)	NR	Female/male Osteoporotic with fracture 68.00 ± 1.00 years Healthy controls 68.10 ± 1.00 years	None	DXA
Zhao 2019 [78]	China	NR	96 Osteoporotic (N = 48) Healthy controls (N = 48)	NR	NR	None	NR
Zhou 2019 [79]	China	NR	144 Osteoporotic (N = 99) Healthy controls (N = 45)	NR	Female Osteoporotic 62.6 ± 3.5 years Healthy controls 42.8 ± 5.5 years	None	DXA

**Table 3:** Demographics data on sarcopenia.

Ref.	Country of Publication	Study Design	Patients Number	Ethnicity	Sex/Age	Comorbidities	Sarcopenia Diagnostic Measures
Chen 2019 b [32]	USA	NR	75 Osteoporotic/osteopenic (N = 46) Sarcopenic (N = 1) Sarco-osteopenic (N = 15) Non-osteoporotic/non-sarcopenic (N = 13)	NR	Female 60–85 years	None	Handgrip dynamometer (grip strength), gait speed, and countermovement jumps
He 2020 [80]	China	NR	186 Sarcopenic (N = 93) Non-sarcopenic (N = 93)	NR	Sarcopenic 76.15 ± 0.58 years Non-sarcopenic 76.19 ± 0.58 years	Hypertension, diabetes mellitus	Appendicular skeletal muscle mass (ASM); relative skeletal muscle mass index (ASM/Ht2)
He 2021 [81]	China	NR	186 Sarcopenic (N = 93) Non-sarcopenic (N = 93)	Ximen Community of Ningbo	Sarcopenic 76.15 ± 0.58 years Non-sarcopenic 76.19 ± 0.58 years	Hypertension, diabetes mellitus	Appendicular skeletal muscle mass (ASM); relative skeletal muscle mass index (ASM/Ht2)
Liu 2021 [82]	China	NR	77 Sarcopenic (N = 18) Dynapenic (loss of muscular function without mass) (N = 35) Non-sarcopenic (N = 24)	Community-dwelling older adults	Female/male Sarcopenic 79.8 ± 5.9 years Dynapenic 80.2 ± 5.7 years Non-sarcopenic 75.8 ± 6.1 years	None	Handgrip strength, gait speed
Valášková 2021 [83]	Slovakia	NR	80 patients classified based on a short physical performance battery score (SPPB): Sarcopenia SPPB ≤ 6 (low muscle performance) (N = 31) Sarcopenia SPPB 7–9 (moderate muscle performance) (N = 17) Sarcopenia SPPB > 9 (high muscle performance) (N = 32)	NR	Female/male 55–86 years	NR	SPPB

**Table 4:** Methodology of studies on osteoporosis.

Ref.	miR	miR Assay	Tissue	Endogenous Control	Technical Replicates	Timing of Sample Collection	miR Direction	Main Results
Al-Rawaf 2021 [26]	miR-148a and miR-122-5p	qRT-PCR	Serum	NR	Triplicate	In the morning, in fasted state	↑ miR-148a ↓ miR-122-5p	↑ miR-148a and ↓ miR-122-5p significantly associated with bone loss or osteoporosis in elderly postmenopausal women
Baloun 2022 [27]	let-7b-5p, miR-320a, miR-375, miR-188-5p, miR-152-3p, miR-582-5p, miR-144-5p, miR-141-3p, miR-127-3p, miR-17-5p	qRT-PCR	Serum	NR	NR	Before oophorectomy/ hysterectomy 201 ± 24 days after surgery 508 ± 127 days after Oophorectomy/hysterectomy 203 ± 71 days after estradiol treatment	No differences	No association of miRs with osteoporosis
Bedene 2016 [28]	let-7d-5p, let-7e-5p, miR-30d-5p, miR-30e-5p, miR-126-3p, miR-148a-3p, miR-199a-3p, miR-423-5p, and miR-574-5p	qRT-PCR	Serum	NR	NR	NR	↑ miR-148a-3p	miR-148a-3p as a potential plasma-based biomarker for osteoporosis
Chen 2016 [29]	miR-30a-5p, miR-30e-5p, miR-425-5p, miR-142-3p, miR-191a-3p, miR-215, miR-29b-3p, miR-30b-5p, miR-26a-5p, miR-345-5p, miR-361-5p, miR-185-5p, miR-103-3p	qRT-PCR	Serum	NR	NR	NR	↓ miR-30b-5p in osteopenia/osteoporosis; ↓ miR-103-3p, miR-142-3p, miR-328-3p in osteoporosis	miR-30b-5p down regulated in postmenopausal women with osteopenia or osteoporosis; ↓miR-103-3p, miR-142-3p, miR-328-3p only in osteoporosis
Chen 2017 [30]	miR-30, miR-96, miR-125b, miR-4665-3p, miR-5914	qRT-PCR	Serum	U6	NR	NR	↑ miR-125b, miR-30, and miR-5914	miR-125b significantly upregulated in postmenopausal osteoporosis
Chen 2019 [31]	miR-19a-3p	qRT-PCR	Serum	U6	NR	In the morning, in fasted state	↓miR-19a-3p	miR-19a-3p down-regulated in osteoporosis
Chen 2019 b [32]	miR-1-3p, miR-21-5p, miR-23a-3p, miR-24-3p, miR-100-5p, miR-125b-5p, miR-133a-3p, miR-206	qRT-PCR	Serum	miR-16-5p, -93-5p, and -191-5p	NR	In the morning, in fasted state	↓ miR-125b-5p and ↑ miR-21-5p and -23a-3 in osteoporosis	Relative expression level of miR-21-5p significantly negatively correlated with trochanter bone mineral content.
Cheng 2019 [33]	miR-365a-3p	qRT-PCR	Serum	NR	NR	In the morning, in fasted state	↑miR-365a-3p	miR-365a-3p highly expressed in osteoporosis
Ciuffi 2022 [34]	miR-8085, miR-320a-3p, miR-23a-3p, miR-4497, miR-145-5p	ddPCR	Serum	Synthetic RNA spike-ins, UniSp2, UniSp4, and UniSp5	NR	NR	↓ miR-23a-3p ↑ miR-320a-3p	Levels of miR-23a-3p and miR-21-5p able to distinguish osteoporotic patients and subjects with normal BMD
Ding 2019 [35]	miR-100	qRT-PCR	Serum	NR	NR	NR	↑ miR-100	miR-100 as potential biomarker for the diagnosis and treatment osteoporosis
Feurer 2019 [36]	miR-133a-3p, miR-20a-5p, miR-25-3p, miR-100-5p, miR-133b, miR-214-3p, miR-26a-5p, miR-103a-3p, miR-145-5p, miR-21-5p, miR-29a-3p, miR-106a-5p, miR-146a-5p, miR-221-5p, miR-29b-3p, miR-122-5p, miR-148a-3p, miR-222-3p, miR-338-3p, miR-124-3p, miR-155-5p, miR-223-5p, miR-34a-5p, miR-125b-5p, miR-17-5p, miR-23a-3p, miR-503-5p, miR-127-3p, miR-204-5p, miR-24-3p, miR-93-5p, miR-16-5p	qRT-PCR	Serum	UniSp6	NR	In the morning, in fasted state	None	No significant association between prevalent or incident fractures, BTM, DXA, and HRpQCT parameters and analyzed miR
Fu 2019 [37]	miR-27a-3p	qRT-PCR	Serum	NR	NR	NR	↓ miR-27a-3p	↓miR-27a-3p in osteoporosis in comparison to controls
Fu 2021 [38]	miR-145-5p	qRT-PCR	Serum	U6	Triplicate	In the morning, in fasted state	↑ miR-145-5p	↑miR-145-5p in osteoporotic in comparison to control
Gao 2020 [39]	miR-217	qRT-PCR	Serum	NR	NR	NR	↑ miR-217	Up-regulation of miR-217 in osteoporotic in comparison to controls
Guo 2022 [40]	miR-221-5p	qRT-PCR	Serum	U6	NR	NR	↓ miR-221-5p	Down-regulation of miR-221-5p in osteoporotic in comparison to controls
Ismail 2020 [41]	miR-208a-3p, miR-155-5p, miR-637	qRT-PCR	Serum	Hs_Snord68_11	Duplicate	For premenopausal women: during the early follicular phase, i.e., days 3–7 of the menstrual cycle	↑ miR-208a-3p, ↓ miR-155-5p	miR-208a-3p significantly upregulated, miR-155-5p markedly down-regulated in the premenopausal patients compared to its respective controls
Li 2014 [42]	miR-21, miR-133a, miR-146a	qRT-PCR	Plasma	miR-16	NR	In the morning, in fasted state	↓ miR-21 ↑ miR-133a	Downregulation of miR-21 and upregulation of miR-133a in osteoporosis and osteopenia patients versus controls
Li 2018 [43]	miR-133a	qRT-PCR	Serum	U6	NR	NR	↑ miR-133a	miR-133a significantly upregulated and negatively correlated with lumbar spine BMD in post-menopausal osteoporotic women
Li 2020 [44]	miR-483-5p	qRT-PCR	Serum	U6	NR	NR	↑ miR-483-5p	↑ expression of miR-483–5p in osteoporotic patients
Lu 2021 [45]	miR-206	qRT-PCR	Serum	U6	NR	NR	↓ miR-206	↓ miR-206 in osteoporotic patient group versus controls
Luo 2019 [46]	miR-579-3p	qRT-PCR	Serum	U6	NR	NR	↑ miR-579-3p	↑ miR-579-3p in osteoporotic patients than controls
Lv 2019 [47]	miR-200a-3p	qRT-PCR	Serum	U6	NR	NR	↑ miR-200a-3p	↑miR 200a-3p in osteoporotic patients relative to controls
Ma 2021 [48]	miR-181c-5p, miR-497-5p, miR-204-3p, miR-1290	qRT-PCR	Serum	5S rRNA	NR	In the morning, in fasted state	↓ miR-181c-5p and miR-497-5p ↑ miR-204-3p	miR-181c-5p and miR-497-5p involved in bone metabolism and associated with progressive bone loss due to osteoporosis
Ma 2022 [49]	miR-455–3p	qRT-PCR	Serum	U6	NR	NR	↓ miR-455–3p	↓ miR-455–3p in osteoporosis and fragility fracture patients compared to controls
Mandourah 2018 [50]	370 mature miRs	qRT-PCR	Plasma and serum	SNORD61, SNORD68, SNORD72, SNORD95, SNORD96A, and RNU6-6P	NR	NR	↓ miR122-5p and miR4516	miR122-5p and miR4516 present at significantly different levels between non-osteoporotic control, osteopenia, and osteoporosis patients
Mi 2020 [51]	miR-194-5p	qRT-PCR	Serum	U6	Triplicate	NR	↑ miR-194-5p	↑ miR-194-5p level linked to osteoporosis
Nakashima 2020 [52]	let7d, miR1, miR17, miR20a, miR21, miR27a, miR34a, miR92, miR103a, miR122, miR126, miR130a, miR133a, miR146, miR150, miR192, miR195, miR197, miR199, miR221, miR222, miR320	qRT-PCR	Serum	NR	NR	In the morning, in fasted state	↓ miR195, ↑ miR150 and miR222	↓ miR195 in osteoporotic females, ↑ miR150 and miR222 in osteoporotic males
Nobrega 2020 [53]	miR-1-3p, miR-21-5p, miR-34a-5p, miR-92a-3p, miR-100-5p, miR-126-3p, miR-130a-3p, miR-146a-5p, miR-155-5p, and miR-221-3p	qRT-PCR	Whole blood	RNU43	NR	In the morning, in fasted state	↑ miR-34a-5p	↑ miR-34a-5p among very old adults who display the lowest scores of BMD
Panach 2015 [54]	Serum/Plasma microRNA PCR Panel	qRT-PCR	Serum	UniSP6 and cel-miR-39	NR	NR	↑ miR-122-5p, miR-125b-5p, and miR-21-5p	miR-122-5p, miR-125b-5p, and miR-21-5p upregulated biomarkers in bone fracture with respect to controls
Pertusa 2021 [55]	miR-497-5p, miR-155-5p, miR-423-5p, miR-365-3p	qRT-PCR	Serum	Cel-miR-39	NR	NR	↑ miR-497 and miR-423 ↓ miR-155 and miR-365	↑ miR-497 and miR-423 and ↓ miR-155 and miR-365 in osteoporotic than in control
Qiao 2019 [56]	miR-203	qRT-PCR	Serum	NR	NR	In fasted state	↓ miR-203	↓ miR-203 in patients with postmenopausal osteoporosis than in controls
Ramírez-Salazar 2019 [57]	miR-23b-3p, miR-140-3p, miR-885-5p	qRT-PCR	Serum	RNU6	NR	NR	↑ miR-140-3p and miR-23b-3p	miR-140-3p and miR-23b-3p as potential biomarkers candidates for osteoporosis
Salman 2021 [58]	miR-133a, miR-25 3p	qRT-PCR	Serum	RNU43	NR	NR	↑ miR-133a	miR-133a as biomarker for osteoporosis
Seeliger 2014 [59]	let-7a-5p, miR-1, miR-100-5p, miR-106b-5p, miR-10b-5p, miR-122-5p, miR-124-3p, miR-125b-5p, miR-126-3p, miR-133a, miR-133b, miR-134, miR-141-3p, miR-143-3p, miR-146a-5p, miR-150-5p, miR-155-5p, miR-17-5p/106a-5p, miR-17-3p, miR-18a-5p, miR-192-5p, miR-195-5p, miR-196a-5p, miR-19a-3p, miR-19b-3p, miR-200a-3p, miR-200b-3p, miR-200c-3p, miR-203a, miR-205-5p, miR-208a, miR-20a-5p, miR-21-5p, miR-210, miR-214-3p, miR-215, miR-221-3p, miR-222-3p, miR-223-3p, miR-224-5p, miR-23a-3p, miR-25-3p	qRT-PCR	Serum	RNU6	Duplicate	NR	↑ miR-21, miR-23a, miR-24, miR-93, miR-100, miR-122a, miR-124a, miR-125b, miR-148a	miR-21, miR-23a, miR-24, miR-93, miR-100, miR-122a, miR-124a, miR-125b, and miR-148a significantly upregulated in the serum of patients with osteoporosis
Shuai 2020 [60]	miR-29b-3p, miR-30c-2-3p, miR-145-5p, miR-199a-5p, miR-301a-3p, miR-497-5p, miR-526b-5p, miR-550a-5p, miR-575, miR-654-5p, miR-877-3p, miR-1260b, miR-4769-3p, miR-15a-5p, miR-424-5p, miR-663a, miR-708-5p, miR-1246, miR-1299, miR-1323, miR-4447, miR-5685	qRT-PCR	Serum	U6	NR	NR	↑ miR-30c-2-3p, miR-497-5p, 550a-5p, miR-654-5p, miR-663a, miR-877-3p, miR-1299 ↓ miR-199a-5p, miR424-5p, miR-1260b	miR-30c-2-3p, miR-199a-5p, miR424-5p, miR-497-5p, miR-550a-5p, miR-654-5p, miR-663a, miR-877-3p, miR-1260b, miR-1299 ere highly expressed in serum and differed significantly among osteopenic, osteoporotic, and healthy patients
Sun 2020a [61]	miR-19b	qRT-PCR	Serum	U6	NR	In the morning, in fasted state	↓ miR-19b	↓ miR-19b in osteoporotic patients with vertebral compression fractures than that in controls
Sun 2020b [62]	miR-211	qRT-PCR	Serum	NR	NR	NR	↑ miR-211	In the fracture group, miR-211 expression was significantly up-regulated compared with controls
Tang 2019 [63]	miR-144	qRT-PCR	Serum	U6	NR	NR	↑ miR-144	Expression of miR-144 upregulated in osteoporotic patients compared with control
Wang 2018 [64]	miR-7-5p miR-211-5p, miR-24-3p, miR-27a-3p, miR-100, miR-125b, miR-122a, miR-128, miR 145, miR-144-3p	qRT-PCR	Serum	NR	Triplicate	NR	↑ miR-24-3p, 27a-3p, 100, 125b, 122a, 145 ↓ miR-144-3p	Significant upregulation of miR-24-3p, 27a-3p, 100, 125b, 145, and 122a in osteoporosis compared to control. miR-144-3p downregulated in in osteoporosis compared to control
Weilner 2015 [65]	miR-10a-5p, miR-10b-5p, miR-133b, miR-22-3p, miR-328-3p, let-7g-5p	qRT-PCR	Serum	U6 and 5S rRNA	NR	Between 8:00 a.m. and 10:00 a.m. in fasted state	↑ miR-22-3p, ↓ miR-328-3p and let-7g-5p	De-regulation of miR-22-3p, miR-328-3p, and let-7g-5p in osteoporotic fractured patients
Wu 2021 [66]	miR-10a-3p	qRT-PCR	Serum	U6	Triplicate	NR	↑ miR-10a-3p	↑ miR-10a-3p in osteoporotic patients
Xia 2018 [67]	miR-203	qRT-PCR	Serum	NR	Triplicate	In the morning, in fasted state	↓ miR-203	↓ miR-203 in osteoporosis patients that in controls
Xu 2022 [68]	miR-491-5p, miR-485-3p	qRT-PCR	Plasma	U6	NR	NR	↓ miR-491-5p and miR-485-3p	Expression levels of miR-491-5p and miR-485-3p declined in osteoporotic patients with vertebral fractures when compared to those without fractures
Yang 2019 [69]	miR-217	qRT-PCR	Serum	NR	NR	In the morning, in fasted state	↑ miR-217	↑ miR-217 in osteoporotic patients
Yavropoulou 2017 [70]	miR-21-5p, miR-23a-3p, miR-24-2-5p, miR-26a-5p, miR-29a, miR-33a-5p, miR-124-3p, miR-135b-5p, miR-214-3p, miR-218-5p, miR-335-3p, miR-2861	qRT-PCR	Serum	RNU6-2	Triplicate	NR	↑ miR-124 and miR-2861; ↓ miR-21, miR-23, miR-29, miR-21-5p	miR-21-5p, miR-23a, miR-29a-3p, miR-124-3p, and miR-2861 significantly deregulated in osteoporotic compared with controls. ↑ miR-124 and miR-2861 and ↓ miR-21, miR-23 and miR-29 in osteoporotic compared with controls. ↓miR-21-5p in osteoporotic/osteopenic women with vertebral fractures
Yin 2022 [71]	miR-215-5p	qRT-PCR	Serum	U6	Triplicate	In fasted state	↓ miR-215-5p	↓ miR-215-5p in patients with osteoporosis
You 2016 [72]	miR-27a	qRT-PCR	Serum	U6	Triplicate	NR	↓ miR-27a	miR-27a significantly down-regulated in postmenopausal osteoporotic patients
Yu 2020 [73]	miR-137	qRT-PCR	Serum	U6	NR	NR	↑ miR-137	↑ miR-137 in osteoporotic in comparison to controls
Yuan 2021 [74]	miR-26a	qRT-PCR	Serum	NR	NR	NR	↑ miR-26a	↑ miR-26a in patients with osteoporosis
Zarecki 2020 [75]	miR-19b-3p miR-486-3p, miR-550a-3p, miR-106b-5p, miR-144-3p, miR-451a, miR-29b-3p, miR-96-5p, miR-188-5p, miR-532-3p, miR-30e-5p, miR-214-3p, miR-143-3p, miR-133b, miR-21-5p, miR-23a-3p, miR-152-3p, miR-335-5p, miR-127-3p, miR-375	qRT-PCR	Serum	cel-miR-39-3p	NR	After an overnight fast	↑ miR-375, miR-532-3p, miR-19b-3p, miR-152-3p, miR-23a-3p, miR-335-5p, miR-21-5p	Up-regulated miR-375, miR-532-3p, miR-19b-3p, miR-152-3p, miR-23a-3p, miR-335-5p, miR-21-5p in patients with vertebral fractures and osteoporosis compared to osteoporosis without fracture and controls
Zhang 2019 [76]	miR-30a-5p	qRT-PCR	Serum	NR	NR	NR	↑ miR-30a-5p	miR-30a-5p significantly upregulated in osteoporosis patients
Zhang 2021 [77]	miR-502-3p	qRT-PCR	Serum	U6	Three duplicates	NR	↓ miR-502-3p	↓ miR-502-3p in osteoporotic than in controls
Zhao 2019 [78]	miR-17, miR-20a, miR-21, miR-26a, miR-29b, and miR-106b	qRT-PCR	Serum	U6	NR	NR	↓ miR-21	↓ miR-21 expression in patients with osteoporosis than in controls
Zhou 2019 [79]	miR-let-7c	qRT-PCR	Serum	NR	Triplicate	NR	↑ miR-let-7c	miR-let-7c up-regulated in patients with postmenopausal osteoporosis compared with controls

↑: increase; and ↓: decrease.

**Table 5:** Methodology of studies on sarcopenia.

Ref.	miR	miR Assay	Tissue	Reference Genes	Technical Replicates	Timing of Sample Collection	miR Direction	Main Results
Chen 2019 b [32]	miR-1-3p, miR-21-5p, miR-23a-3p, miR-24-3p, miR-100-5p, miR-125b-5p, miR-133a-3p, miR-206	qRT-PCR	Serum	miR-16-5p, miR-93-5p, miR-191-5p	NR	In the early morning after overnight fasting	None	The study did not determine specific circulating miRs as biomarkers for sarcopenia
He 2020 [80]	miR-155, miR-208b, miR-222, miR-210, miR-328, miR-499, mir-133a, miR-133b, miR-21, miR-146a, miR-126, miR-221, and miR-20a	qRT-PCR	Plasma	cel-miR-39	NR	After overnight fasting	↓ miR-155, miR-208b, miR-222, miR-210, miR-328, and miR-499	miR-155, miR-208b, miR-222, miR-210, miR-328, and miR-499 significantly down-regulated in sarcopenic patients compared to non-sarcopenic
He 2021 [81]	miR-637, miR-148a-3p, miR-125b-5p, miR-124-3p, miR-122-5p, miR-100-5p, miR-93-5p, miR-21-5p, miR-23a-3p, and miR-24-3p	qRT-PCR	Plasma	cel-miR-39	NR	After overnight fasting	↓ miR-23a-3p, miR-93-5p, and miR-637	↓ miR-23a-3p, miR-93-5p, miR-637 in the sarcopenia group than in the non-sarcopenia group
Liu 2021 [82]	miR-133a, miR-486, miR-21, miR-146a	qRT-PCR	Plasma	cel-miR-39-3p	NR	Fasting for at least 8 h and avoidance of strenuous physical exercise for at least 48 h	↓ miR-486 and miR-146a	Myo-miR (miR-486) and inflammation-related miR (miR-146a) as biomarkers of age-related sarcopenia
Valášková 2021 [83]	miR-29a, miR-29b, miR-1, miR-133a, miR-133b, miR-206, miR-208b and miR-499	qRT-PCR	Plasma	ce-miR-39	NR	NR	↑ miR-1, miR-29a and miR-29b; ↓ miR-206, miR-133a, miR-133b, miR-208b, and miR-499	↑ miR-1, miR-29a, and miR-29b and ↓miR-206, miR-133a, miR-133b, miR-208b, and miR-499 expression in patients with low muscle performance

↑: increase; and ↓: decrease.

## 3. Results

### 3.1. Study Selection and Characteristics 

**Figure 2:**
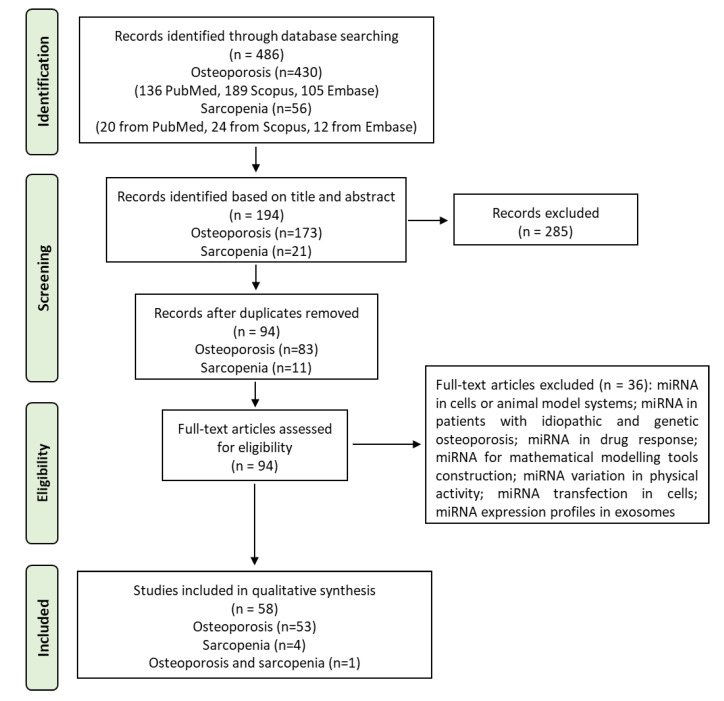
The PRISMA flow diagram for the systematic review detailing the database searches, the number of abstracts screened, and the full texts retrieved.

The initial literature search retrieved 486 studies. Of those, 430 studies (136 from PubMed, 189 from Scopus, 105 from Embase) were on osteoporosis and 56 were on sarcopenia (20 from PubMed, 24 from Scopus, 12 from Embase). Articles were screened for title and abstract, and 194 articles were selected: 173 for osteoporosis and 21 for sarcopenia. Subsequently, these articles were submitted to a public reference manager to eliminate duplicates. The resulting 94 complete articles, 83 on osteoporosis and 11 on sarcopenia, were then reviewed to establish whether the publications met the inclusion criteria, and 58 (53 on osteoporosis, 4 on sarcopenia, and 1 on both osteoporosis and sarcopenia) studies were considered eligible for this review. The search strategy and study inclusion and exclusion criteria are detailed in Figure 2.

### 3.2. Study General Characteristics

Table 2 and Table 4 describe the study demographics characteristics respectively for osteoporosis and sarcopenia. Most studies (69%) on osteoporosis do not define the study design; studies where the types of cohorts are specified are prospective (n = 10), case-control, and/or cross-sectional (n = 6) and retrospective (n = 1). None of the studies on sarcopenia defined the study design. Most of the studies (68%) were conducted in China, but participant ethnicity is stated in very few studies (25%) [29,30,35,42,43,52,53,54,55,57,60,65,81,82], thus limiting the generalizability of findings.

For osteoporosis, the largest cohort included 682 patients (99 pre-menopausal woman and 583 post-menopausal woman) [36]. Furthermore, 23/54 (43%) studies on osteoporosis had patient cohorts ≥ 100 subjects, while all the others had smaller patient cohorts, with the smallest cohort including 18 patients (6 with osteoporotic fracture, 6 osteoporotic without fractures, and 6 healthy controls) [61]. Additionally, 3 of 54 studies did not specify the total number of patients recruited for the study. For sarcopenia, the largest cohort included 186 patients (93 sarcopenic and 93 non-sarcopenic) [80,81], while the smallest included 65 patients [32]. Moreover, 85% of studies on osteoporosis (46/54) had a healthy control group to compare osteoporotic/osteopenic fractured and/or non-fractured groups. Osteoporosis was diagnosed by DXA in ~65% of the studies (37/54 studies), which was sometimes also associated to pQCT, X-ray, physical examination, and FRAX tool [28,36,59,67]. Concerning sarcopenia, it was diagnosed by SPPB score, appendicular skeletal muscle mass (ASM) analysis, relative skeletal muscle mass index, and by grip strength, gait speed, and countermovement jumps tests [32,80,81,82,83]. The most common age group for osteoporosis, sarcopenia, and healthy controls was 60–75 years. Some studies (17%) recruited younger participants in healthy control groups [38,41,42,44,47,59,60,71,78] and did not match for age, causing potential selection bias. Almost all the studies considered female osteoporotic and sarcopenic patients (76%), but 14/59 studies also included male patients [34,38,50,52,53,58,59,60,61,62,66,73,77,82,83]. Twelve studies on osteoporosis and two on sarcopenia did not state sex.

### 3.3. miRs Dysregulation in Osteoporosis and Sarcopenia

As reported in Table 4 and Table 5, differential miRs expression is defined as an alteration, i.e., up- or down-regulation, both in osteoporosis/osteopenia and sarcopenia versus healthy controls, including statistically significant *p*-values < 0.05. In studies that reported a discovery phase and validation phase, only miRs confirmed in the validation phase were considered for this review. 

### 3.4. miRs in Osteoporosis 

In this review, more than 69 circulating miRs were dysregulated and differentially expressed in osteoporosis, but the most widely dysregulated was miR-21 (primarily the -5p form), with n = 7 studies (12.7%), followed by miR-23 with n = 6 studies (10.9%), miR-122, and miR-125b with n = 5 studies each (9% each), miR-27 and miR-30 with n = 4 studies (7.2%), and miR-19, miR-148, miR-100, miR-497, miR-24 and miR-133a or b with three studies each (5.4% each). All other miRNAs were considered in two or one studies (miR-320a-3p, miR-103-3p, miR-142-3p, miR-221-5p, miR-208a-3p, miR-483-5p, miR-206, miR-579-3p, miR 200a-3p, miR-181c-5p, miR-204-3p, miR4516, miR-455–3p, miR-194-5p, miR-150, miR-222, miR-195, miR-34a-5p, miR-140-3p, miR-423, miR-93, miR-1299, miR-550a-5p, miR-654-5p, miR-663a, miR-877-3p, miR-199a-5p, miR-424-5p, miR-1260b, miR-211, miR-22-3p, miR-let-7g-5p, miR-10a-3p, miR-491-5p, miR-485-3p, miR-2861, miR-29, miR-215-5p, miR-137, miR-26a, miR-375, miR-532-3p, miR-335-5p, miR-152-3p, miR-502-3p, miR-let-7c).

For miR-21, not all studies agreed on direction of expression, with four studies reporting up-regulation [32,54,59,75] and 3 down-regulation [42,70,78] in the osteoporotic groups compared to the non-osteoporotic groups. MiR-21 up-regulation and down-regulation was described also for osteoporotic fracture patients [54,75,78]. The study by Li et al. [42] also showed down-regulation of miR-21 in plasma from osteopenic patients; a Greek study [70] showed downregulation of miR-21 and miR-21-5p in serum of patients with low BMD and vertebral fracture in comparison to patients with low BMD and no fracture.

MiR-23a and its -3p form were up-regulated in four studies [32,57,59,75] and down-regulated in two studies [34,70]. Ciuffi et al. [34], in their prospective multicenter study, measured miRs by using a next-generation sequencing -based prescreening profile approach considering not only female patients but also osteopenic and/or osteoporosis male patients, showing the deregulated serum levels of miR-23a-3p in osteoporotic patients as well as their relationship with bone quality parameters and sensitivity/specificity in distinguishing osteoporotic patients from normal BMD subjects. Yavropoulou et al. [70] also showed deregulated serum level of miR-23a in patients with low bone mass compared with healthy controls. Differently, up-regulation of miR-23a was associated with BMD variation and vertebral fracture in other studies [32,57,59,75].

Concerning miR-122 [26,50,54,59,64] and miR-125 [30,32,54,59,64], their expression level in the different studies were conflicting for direction of regulation. In 3/5 studies, miR-122 was upregulated [54,59,64] in osteoporotic patients versus healthy controls, while in the remaining studies [26,50], it was downregulated. In particular, Mandourah et al. [50] showed that miR-122 was significantly differentially expressed between non-osteoporotic controls, osteopenic, and osteoporotic patients. Concerning miR-125b, it was overexpressed in almost all the studies (4/5), except for the study by Chen et al. [32], wherein a downregulation in the osteoporotic group compared to the non-osteoporotic group was seen. A similar trend was seen also for miR-30 [29,30,60,76] that resulted in overexpression in 3/4 studies. Only one study, by Chen et al. [29], revealed that miR-30b-5p was significantly down-regulated in postmenopausal women with osteopenia or osteoporosis. In contrast, miR-27a and its -3p form was down-regulated in postmenopausal women with osteoporosis in comparison to healthy controls in almost all the studies [37,72] and up-regulated in only one study [64].

Studies on miR-148 [26,28,59], miR-133a or b [42,43,58], and miR-100 [35,59,64] agreed for upregulation of these miRs, while conflicting results for direction of regulation were seen for miR-497 [48,55,60], miR-19a or b [31,61,75], and miR-24 [59,64].

Other microRNAs with agreement on the direction of change in osteoporosis included miR-124 [59,70], miR-145 [38,64] (up-regulation) and miR-155 [40,55], miR-203 [56,67], miR-206 [45,72], miR-328 [29,65] (down-regulation). Moreover, miR-208a was evaluated in two studies [41,59], but its isoform 3p was up-regulated in serum from osteoporotic patients in only one study [41]. Similarly, miR-222 [36,52] and miR-93 [36,59] were also evaluated in two studies, but they were up-regulated only in one [52,59]. Finally, miR-93a or b was evaluated in five studies [36,60,75,78], but it was down-regulated in osteoporotic patients only in two of them [70,72].

### 3.5. miRs in Sarcopenia

In this review, 14 circulating miRs were dysregulated and differentially expressed in sarcopenia (miR-206, miR-208b, miR-222, miR-210, miR-328, miR-93-5p, miR-146a, miR-155, miR-23a-3p, miR-486, miR-1, miR-29, miR-133a and b, miR-499).

Chen et al. [32], examining miR-1-3p, -21-5p, -23a-3p, -24-3p, -100-5p, -125b-5p, -133a-3p, and -206 in the serum of 65 patients, did not find a specific alteration of circulating miRs. In contrast to the study of Chen et al. [32], other studies found that patients with low muscle performance (sarcopenic) showed increased expression of miR-1, miR-29a, and miR-29b, but also a decreased expression of miR-486, miR-146a, miR-206, miR-133a, miR-133b, miR-208b, and miR-499 [82,83]. Alteration in circulating miRs was also demonstrated by He et al.: examining plasma from sarcopenic and non-sarcopenic patients showed that miR-155, miR-208b, miR-222, miR-210, miR-328, and miR-499 were significantly down-regulated in sarcopenic compared to non-sarcopenic patients [80]. Finally, they also revealed that the relative expression levels of plasma miR-23a-3p, miR-93-5p, and miR-637 in the sarcopenic group were significantly lower than that in the non-sarcopenia group [81].

### 3.6. Sharing miRs between Osteoporosis and Sarcopenia

**Figure 3:**
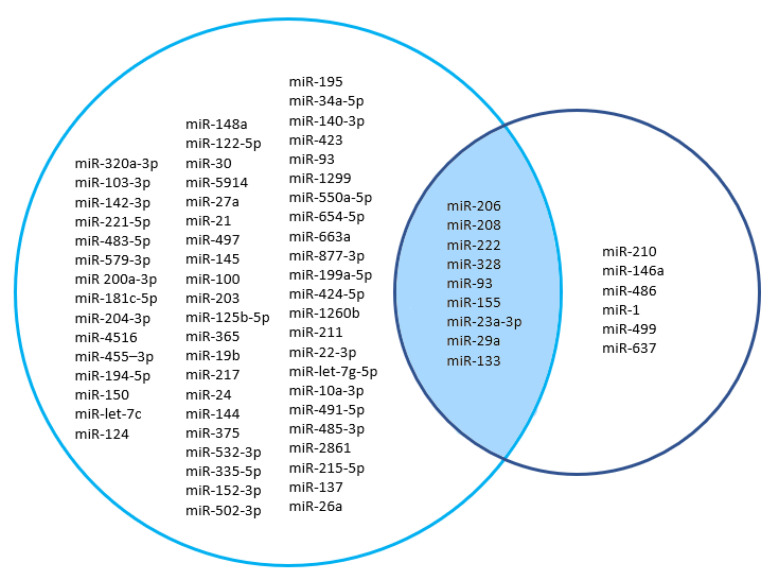
Shared miRs between osteoporosis and sarcopenia.

Between osteoporosis and sarcopenia, there was a moderate degree of overlap of dysregulated miRs. Specifically, nine shared miRs between osteoporosis and sarcopenia were detected in this review: miR-206, miR-208, miR-222, miR-328, miR-93, miR-155, miR-23a-3p, miR-29a, and miR-133a and b (Figure 3). However, for most of these miRs, there has been no replication by more than one study, and this is particularly true for all miRs analyzed in sarcopenia. In contrast, for osteoporosis, miR-222, miR-23a, and miR-133a or b were respectively found in five, four and three studies.

### 3.7. Risk of Bias Assessment

More than 60% of the studies on osteoporosis included in the current systematic review satisfied most of the items in the QUADAS2, which suggests that the overall quality of included studies was moderate-to-high (Table 6). Six studies showed a high risk of bias in the patient selection [29,32,39,46,53,76] by not reporting, beyond the type of study, the allocation of the number of patients in the different experimental groups. Concerning the index text and how it was conducted and/or interpreted, most of the included studies showed a low risk of bias. However, several studies did not specify the endogenous control used to perform the qRT-PCR, and thus they were scored to have a high risk of bias in relation to the index test [29,33,35,37,39,53,57,62,64,67,69,74,76,79]. The reference standard and the flow and timing risk of bias were low in all the examined studies.

All the studies on sarcopenia [32,81,82,83] satisfied all the items in the QUADAS2, which suggests that the overall quality of the included studies was high.

## 4. Discussion

Osteosarcopenia is a complex and multifactorial disabling disease that is characterized by decreasing bone and muscle mass that is often followed by low-traumatic fracture occurrences and muscle atrophy with a strong negative impact on the quality of life and important socio-economic repercussions [8,9,10,11]. The availability of valid diagnostic tools to identify the onset, progression, and manifestation of osteoporosis has allowed physicians to manage the pathological condition more effectively. However, osteoporosis tools are not yet able to guarantee the necessary sensitivity and specificity [13,14]. Concerning sarcopenia, because of confused definitions and inaccurate screening tools, it frequently remains undiagnosed [13,14]. Osteosarcopenia, which identifies the concomitant presence of sarcopenia and osteoporosis, does not have a unique model of diagnosis but is based on the reference definitions of osteoporosis and sarcopenia, which at present still have limitations. However, if a diagnosis of sarcopenia according to the indications of EWGSOP2 is present, a low bone mass determined by the T-score BMD confirms a diagnosis of osteosarcopenia [8]. This diagnostic criterion was further confirmed by Tarantino et al. in a recent meta-analysis that identified a new potential predictive model based on the correlation of T-score and handgrip strength. The results of this study confirmed how the trend of these variables goes hand-in-hand with the progressive increase in the severity of the osteoporotic and sarcopenic condition, up to osteosarcopenia [84]. However, in addition to imaging modalities and tools for osteosarcopenia diagnosis, the biochemical assessment of bone and muscle metabolism has been also proposed to improve early diagnosis and screening. Furthermore, in recent years, the scientific community has focused its attention on a novel class of potential diagnostic biomarkers, both for osteoporosis and sarcopenia, named circulating cell-free miRs [15,16,17,18,19,20]. Several studies have shown that miRs in cultured cells or animal models may play pivotal roles in osteoporosis and sarcopenia, but fewer data are available on circulating miRs [15,16,17,18,19,20,21]. Thus, given the increasing prevalence of osteosarcopenia in elderly populations, we systematically evaluated the potential clinical biomarker utility of circulating miRs in patients with a diagnosis of osteoporosis and sarcopenia versus healthy controls and evaluated the shared miRs between these two pathological conditions. Th results of this review show that more than 69 circulating miRs were dysregulated and differentially expressed in osteoporosis, while only 14 miRs were dysregulated in sarcopenia. The small number of studies on sarcopenia is probably due to the variety of operational definitions used for diagnosis. Even in the studies included in this review, the diagnosis of sarcopenia was not clear, with sarcopenic parameters measured but not used to form a definite diagnosis. However, despite this, our review founded a moderate degree of overlap of dysregulated miRs between osteoporosis and sarcopenia, and this was probably due to the common factors shared between the pathological conditions, e.g., DNA damage, stem-cell depletion, and oxidative stress [8]. 

Ultimately, we identified nine shared miRs that are differentially expressed both in sarcopenia and osteoporosis. These findings are particularly novel, as miRs have not yet been explored in the context of osteosarcopenia syndrome. In this review, it was shown that the shared miRs between osteoporosis and sarcopenia were miR-23a-3p, miR-29a, miR-93, miR-133a, miR-155, miR-206, miR-208, miR-222, and miR-328. However, most of these shared miRs do not exhibit the same direction of dysregulation in osteoporosis and sarcopenia. Only miR-155, miR-206, and miR-328 showed the same dysregulation (down-regulation) in both osteoporosis and sarcopenia. Furthermore, for most of the shared miRs found in this review, there has been no replication by more than one study, particularly for miRs analyzed in sarcopenic patients, while for osteoporosis, three shared miRs, i.e., miR-222, miR-23a, and miR-133a, were found in multiple studies. 

MiR-133a is one of the most studied and best characterized miRs [85,86]. Specifically expressed in muscles, it has been categorized as myomiRs and is essential for appropriate skeletal muscle development and function. In addition to its role in muscle, various studies highlighted that miR-133a can also increase osteoclastogenesis due to mRNA targeting of the proteins that inhibit osteoclastogenesis [85]. In fact, it targets the RUNX2 gene 3′-UTR, a transcription factor indicated as a master regulator in the commitment to the osteoblastic cell line: when this miRNA is overexpressed, it showed a suppression of alkaline phosphatase (ALP) (a marker of osteoblast formation) production and, therefore, osteoblast differentiation [86]. Other muscle-specific miRs are represented by miR-206, miR-208, and miR-222, this last miR being critical for the process of myogenesis and homoeostasis of skeletal muscle [87,88,89,90]. Although miR-222 is clearly important for muscle cell development, the mechanisms by which it regulates myogenesis are still poorly defined. This is in part because the complete set of this miR targets is not known. Despite its roles in muscle, several studies suggested that miR-222 also plays a significant role in vascular formation, which is an essential part of fracture healing [91]. In this context, another miR associated with osteoporotic fracture is the miR-23a-3p, which is associated with osteogenic differentiation and is downregulated in patients with osteoporotic fractures [92]. Moreover, this miR also plays important roles in the myogenesis of skeletal muscle, fiber type determination, and exercise adaptation. In fact, it was shown that the overexpression of miR-23-3p could suppress muscle atrophy both in vitro and in vivo [93].

For some of the shared miRs in this review, there were limited studies in the context of both osteoporosis and sarcopenia, and therefore, their relevance is even less clear at present. From this perspective, even if miR-93 represents the most significantly downregulated miR during osteoblast mineralization [94], only one study on its expression was found for osteoporosis as well as for sarcopenia. Similarly, miR-29, which is implicated in mammalian osteoblast differentiation targeting extracellular matrix molecules and modulating Wnt signaling and regulators of fibrogenesis in muscle targeting ECM proteins such as collagens, fibrillins, and elastin [95,96,97,98,99,100,101], was studied in only one study for both osteoporosis and sarcopenia. Another shared miR between osteoporosis and sarcopenia is represented by miR-155. Wu et al. showed that suppressing the expression and function of this miR contributes to mitigating the inhibition of tumor necrosis factor (TNF)-α on bone morphogenetic protein (BMP)-2-induced osteogenic differentiation [95], indicating that there was a link between miR-155 and BMP signaling. Furthermore, this study also demonstrated that miR-155 facilitates skeletal muscle regeneration by balancing pro- and anti-inflammatory macrophages [102].

While this review followed the Cochrane approach in conducting a systematic review and used an authenticated tool for risk of bias (QUADAS2), achieving excellent inter-rater agreement and conducting screening and risk of bias assessments using more than one reviewer, several limitations must be considered. First, the heterogeneity of the studies identified in this review must be recognized. It is well-known that age affects miRs profiles; thus, older osteoporotic patients could have different miR profiles than younger post-menopausal osteoporotic patients. Similarly, men and women may display differing profiles within the same condition. Second, in several of the included studies, a poor selection of controls within and improper choice of diagnostic criteria, especially for sarcopenia, were present. Third, details about selection procedures and participant demographics were in some cases vague, sample sizes were small, and technical aspects of quality assurance were sometimes omitted.

## 5. Conclusions

This is the first review to examine the potential role of miRs in the context of osteosarcopenia syndrome, thus offering a new perspective on this topic. Here, we provided a complete overview of this topic and identified a panel of miRs that may be involved in osteosarcopenia. Considering the synergistic effect of osteoporosis and sarcopenia on the risk of adverse health outcomes (falls, hospitalization, worsening disability, and all-cause mortality), understanding the pathogenesis of osteosarcopenia syndrome has the potential to lead to effective screening, monitoring, or treatment strategies. However, this systematic review was primarily exploratory, and further research is required to validate the presented findings.

## Figures and Tables

**Table 1:** Combination of free-vocabulary and/or Medical Subject Headings (MeSH) terms for the identification of studies in PubMed, Scopus, and Web of Science.

PubMed	
Osteoporosis	((“osteoporosis” [MeSH Terms] OR “osteoporosis” [All Fields] OR “osteoporoses” [All Fields] OR “osteoporosis, postmenopausal” [MeSH Terms] OR (“osteoporosis” [All Fields] AND “postmenopausal” [All Fields]) OR “postmenopausal osteoporosis” [All Fields]) AND (((“serum” [MeSH Terms] OR “serum” [All Fields] OR “serums” [All Fields] OR “serum s” [All Fields] OR “serumal” [All Fields]) AND (“microrna s” [All Fields] OR “micrornas” [MeSH Terms] OR “micrornas” [All Fields] OR “microrna” [All Fields])) OR (“circulating microrna” [MeSH Terms] OR (“circulating” [All Fields] AND “microrna” [All Fields]) OR “circulating microrna” [All Fields]))) AND ((y_10[Filter]) AND (fha[Filter]) AND (humans[Filter]) AND (english[Filter]))
Sarcopenia	((“sarcopenia” [MeSH Terms] OR “sarcopenia” [All Fields] OR “sarcopenia s” [All Fields]) AND (((“serum” [MeSH Terms] OR “serum” [All Fields] OR “serums” [All Fields] OR “serum s” [All Fields] OR “serumal” [All Fields]) AND (“microrna s” [All Fields] OR “micrornas” [MeSH Terms] OR “micrornas” [All Fields] OR “microrna” [All Fields])) OR (“circulating microrna” [MeSH Terms] OR (“circulating” [All Fields] AND “microrna” [All Fields]) OR “circulating microrna” [All Fields])) AND “2013/01/02 00:00”:”3000/01/01 05:00” [Date—Publication]) AND ((y_10[Filter]) AND (fha[Filter]) AND (humans[Filter]) AND (english[Filter]))
Scopus	
Osteoporosis	(TITLE-ABS-KEY(osteoporosis)) AND (TITLE-ABS-KEY (serum AND microrna) OR TITLE-ABS-KEY (circulating AND microrna)) AND (PUBYEAR > 2011) AND (LIMIT-TO (DOCTYPE,”ar”)) AND (LIMIT-TO (LANGUAGE,”English”))
Sarcopenia	(TITLE-ABS-KEY (sarcopenia) AND TITLE-ABS-KEY (serum AND microrna) OR TITLE-ABS-KEY (circulating AND microrna)) AND PUBYEAR > 2012 AND (LIMIT-TO (DOCTYPE, “ar”)) AND (LIMIT-TO (LANGUAGE, “English”))
EMBASE	
Osteoporosis	(‘osteoporosis’/exp OR osteoporosis) AND (‘serum microrna’ OR ((‘serum’/exp OR serum) AND (‘microrna’/exp OR microrna)) OR ‘circulating microrna’/exp OR ‘circulating microrna’ OR (circulating AND (‘microrna’/exp OR microrna))) AND [2013–2023]/py AND [humans]/lim AND [abstracts]/lim AND [clinical study]/lim AND [embase]/lim AND [article]/lim AND [english]/lim
Sarcopenia	(‘sarcopenia’/exp OR sarcopenia) AND (‘serum microrna’ OR ((‘serum’/exp OR serum) AND (‘microrna’/exp OR microrna)) OR ‘circulating microrna’/exp OR ‘circulating microrna’ OR (circulating AND (‘microrna’/exp OR microrna))) AND [humans]/lim AND [abstracts]/lim AND [clinical study]/lim AND [embase]/lim AND [2013–2023]/py AND [article]/lim AND [english]/lim

**Table 6:** Summary of risk-of-bias assessment (QUADAS-2 tool). Green: low risk of bias or low concern in applicability. Orange: unclear risk. Red: high risk of bias or high concern in applicability. The assessment is weighted based on the sample size in each study.

	Risk of Bias					Applicability Concerns		
	Patients Selection	Index Test	References Standard	Flow and Timing		Patients Selection	Index Test	References Standard
Al-Rawaf 2021 [26]								
Baloun 2022 [27]								
Bedene 2016 [28]								
Chen 2016 [29]								
Chen 2017 [30]								
Chen 2019 [31]								
Chen 2019 b [32]								
Cheng 2019 [33]								
Ciuffi 2022 [34]								
Ding 2019 [35]								
Feurer 2019 [36]								
Fu 2019 [37]								
Fu 2021 [38]								
Gao 2020 [39]								
Guo 2022 [40]								
Ismail 2020 [41]								
Li 2014 [42]								
Li 2018 [43]								
Li 2020 [44]								
Lu 2021 [45]								
Luo 2019 [46]								
Lv 2019 [47]								
Ma 2021 [48]								
Ma 2022 [49]								
Mandourah 2018 [50]								
Mi 2020 [51]								
Nakashima 2020 [52]								
Nobrega 2020 [53]								
Panach 2015 [54]								
Pertusa 2021 [55]								
Qiao 2019 [56]								
Ramírez-Salazar 2019 [57]								
Salman 2021 [58]								
Seeliger 2014 59]								
Shuai 2020 [60]								
Sun 2020a [61]								
Sun 2020b [62]								
Tang 2019 [63]								
Wang 2018 [64]								
Weilner 2015 [65]								
Wu 2021 [66]								
Xia 2018 [67]								
Xu 2022 [68]								
Yang 2019 [69]								
Yavropoulou 2017 [70]								
Yin 2022 [71]								
You 2016 [72]								
Yu 2020 [73]								
Yuan 2021 [74]								
Zarecki 2020 [75]								
Zhang 2019 [76]								
Zhang 2021 [77]								
Zhao 2019 [78]								
Zhou 2019 [79]								
He 2020 [80]								
He 2021 [82]								
Liu 2021 [82]								
Valášková 2021 [83]								

## Data Availability

Not applicable.
